# Correlation between strength/endurance of paraspinal muscles and sagittal parameters in patients with degenerative spinal deformity

**DOI:** 10.1186/s12891-023-06747-6

**Published:** 2023-08-10

**Authors:** Can Chen, Sen Yang, Yong Tang, Xueke Yu, Chunhua Chen, Chengmin Zhang, Fei Luo

**Affiliations:** 1 Department of Orthopaedics, Southwest Hospital, Army Medical University (Third Military Medical University), No 30, Gaotanyan Street, 400038 Shapingba, Chongqing, China; 2 Department for Combat Casualty Care Training, Training Base for Army Health Care, Army Medical University (Third Military Medical University), 400038 Chongqing, China; 3 Department of Orthopaedics, The Hospital of Eighty-third Army, Xinxiang Medical College, 210 Wenhua Street, Hongqi District, 453000 Xinxiang, Henan province China

**Keywords:** Correlation, Strength, Endurance, Paraspinal muscles, Sagittal parameters

## Abstract

**Background:**

Sagittal imbalance is a common cause of low back pain and dysfunction in patients with degenerative spinal deformity (DSD), which greatly affects their quality of life. Strength and endurance are important functional physical indexes for assessing muscle condition. However, the correlation between sagittal parameters and paraspinal muscle strength/endurance is not yet clear. The purpose of this study was to analyze the correlation between strength/endurance of paraspinal muscles and sagittal parameters in patients with DSD.

**Methods:**

There were 105 patients with DSD and 52 healthy volunteers (control group) enrolled. They were divided into the balance group [sagittal vertical axis (SVA) < 5 cm, n = 68] and imbalance group (SVA ≥ 5 cm, n = 37). The maximal voluntary exertion (MVE)/Endurance time (ET) of paravertebral muscles were assessed using the prone position test stand, and the sagittal parameters of the subjects were measured, namely, SVA, thoracic kyphosis (TK), lumbar lordosis (LL), pelvic incidence (PI), pelvic tilt (PT), and sacral slope (SS). Pearson coefficients were used to assess the correlation between paraspinal muscle MVE/ET and sagittal parameters.

**Results:**

MVE and ET of paravertebral muscles in the control group were significantly higher than those in the balance and imbalance groups (P < 0.05), whereas MVE in the balance group was significantly higher than that in the imbalance group (P < 0.05). SVA in the imbalance group was significantly higher than those in the control and balance groups (P < 0.05). SS and TK in the control group were significantly higher than those in the imbalance group (P < 0.05), and PT and PI in the control group were significantly lower than those in the balance and imbalance groups (P < 0.05). LL in the imbalance group was significantly lower than that in the balance and control groups (P < 0.05). MVE, MVE/BH, and MVE/BW of paraspinal muscles in the imbalance group were negatively correlated with SVA and PT. Moreover, they were positively correlated with LL.

**Conclusions:**

Deformity may cause the decrease of MVE and ET of paraspinal muscles in the prone position in patients with DSD. Furthermore, the decline in MVE of paraspinal muscles may be a predisposing factor for the imbalance observed. The decrease of MVE/BW of paraspinal muscles may be involved in spinal compensation, and it is a sensitive indicator for sagittal imbalance and lumbar lordosis.

## Background

The incidence of degenerative spinal deformity (DSD) in the middle-aged and elderly population is increasing annually worldwide [1]. Sagittal parameters are currently recognized as a key factor in predicting surgical outcomes and evaluating the risk of revision [2]. Sagittal imbalance is a common cause of low back pain and dysfunction in patients with DSD, in turn affecting their quality of life [3]. Therefore, preventing the onset of sagittal imbalance is crucial to improve both the diagnosis and treatment of DSD.

Paraspinal muscles are one of the key muscles maintaining the stability and dynamic balance of the spine [4], which play an important role in walking and maintaining upright posture [5], and their dysfunction is related to the occurrence and development of lower back pain, functional impairment, and DSD in the middle-aged and elderly [6, 7]. However, as important as the evaluation indexes of muscle functional physics are, including the maximal voluntary exertion (MVE) and endurance time (ET) of paraspinal muscle [8], there is still a lack of recognized, accurate, and objective evaluation methods. The hand-held muscle strength evaluation based on external fixation test frame has been shown to have good reliability in previous reports [9]. It has been widely used in muscle strength evaluation of paravertebral muscles and hip and knee joints. In addition, the prone position endurance test can effectively reduce lumbar lordosis and avoid lumbar hyperextension, and it is characterized by safety and convenience. Currently, the correlation between paraspinal muscle strength/endurance and sagittal parameters is not clear.

The objectives of this study were (1) to investigate the characteristics of paraspinal muscle MVE/ET degeneration in the prone position, (2) to assess the correlation between paraspinal muscle MVE/ET and sagittal parameters in patients with DSD, and (3) to provide a reference for exploring the pathogenesis of degenerative spinal deformities and guiding clinical diagnosis and treatment.

## Methods

### Participants

Patients with DSD and healthy volunteers were recruited from Southwest Hospital (Chongqing, China) from September 2018 to December 2022. This study has been approved by the Ethics Committee of Southwest Hospital of Army Medical University (Approval Letter No. KY2020235), and all of the participants signed the informed consent form.

The inclusion criteria were as follows: **Balance group**: (1) patients diagnosed with DSD (SVA < 5 cm and Cobb angle on the coronal plane > 10°) and (2) those aged ≥ 45 years, regardless of gender; **Imbalance group**: (1) patients diagnosed with DSD (SVA ≥ 5 cm and/or Cobb angle on the coronal plane > 10°) and (2) those aged ≥ 45 years, regardless of gender; and **Control group**: Subjects with normal spine morphology and aged ≥ 45 years old, regardless of gender.

The exclusion criteria were as follows: (1) Subjects with a history of spinal disease, including congenital spinal disease, spinal tuberculosis, and spinal tumor; (2) those with a history of spinal surgery within the last 2 years or a history of spinal compression fracture within the last 1 year; (3) those with severe hip and knee joint disease or other serious system diseases; (4) those with obvious symptoms of current low back pain that is impacting their daily life; or (5) those who received physical therapy or strength/endurance training for the paraspinal muscles in the past six months.

### MVE/ET test of paraspinal muscles in prone position

**Fig. 1:**
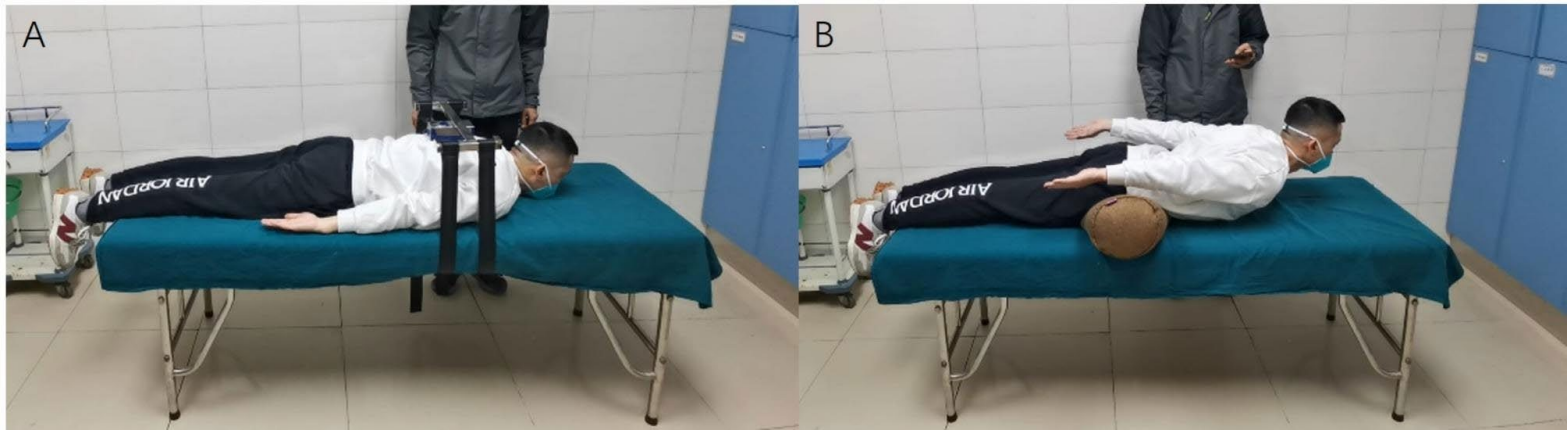
Function assessment of paraspinal muscles. Illustration: (**A**) MVE test of paraspinal muscles in the prone position. (**B**) ET test of paraspinal muscles in the prone position. MVE, maximal voluntary exertion; ET, endurance time

The paraspinal muscle MVE test was conducted as follows. (1) The digital muscle strength tester (MicroFET3) was installed on the support of paraspinal muscle strength test in the prone position. (2) The subject lay in a prone position on the testbed with both feet beyond the back edge of the bed [9] (Fig. 1). (3) The test bracket was fixed on the subject, and the center of the force pad was adjusted so that it was located at the midpoint of the line connecting the superior angles of the scapula on both sides [10]. (4) At the beginning of the test, the subject was asked to do a back extension with maximum strength while simultaneously raising their upper arm, maintaining this position for about 3–5 s and then relaxing, at which time the value on the muscle strength meter was recorded. (5) Three measurements were recorded in total, and the maximum value was taken as the final result.

The paravertebral muscle ET test was conducted as follows. (1) The subject was asked to lie in the prone position on the testbed, and a soft pillow was placed on their lower abdomen to make the midpoint of the iliac crest line basically consistent with the center of the pillow. (2) The subject was asked to exert force upward to do a back extension, placing both upper arms on both sides of their body while simultaneously lifting their sternum away from the surface of the bed, and maintaining the angle between the upper body plane and the surface of the bed at about 15°. The subject was asked to try to maintain this posture during the test [11] (Fig. 1). (3) The timer was stopped and the total time taken when either the angle between the upper body and the surface of the bed was less than 5°, or the subject stayed on the surface of the bed under their sternum for 3 consecutive times, or they failed to tolerate the motion.

### Sagittal parameter measurement

All of the subjects were evaluated for full length spine radiographs. Sagittal parameters were measured by two spine surgeons with  > 5 years of experience. The mean value of the two measurements was taken as the final result. The sagittal parameters that were measured were: (1) sagittal vertical axis (SVA); (2) sacral slope (SS); pelvic tilt (PT); pelvic incidence (PI); (5) lumbar lordosis (LL); and thoracic kyphosis (TK) [12].

### Statistical analysis

Statistical analysis of data was performed using SPSS 25.0 (Chinese version), and the measurement data were described by ($\bar X$ ± s). One-way ANOVA or Kruskal-Wallis H analysis was used for intergroup comparison. *Chi*-square test was used for the constituent ratio among groups. Pearson correlation coefficient was used to assess the correlation between MVE/ET of paravertebral muscles (including those after weighted correction of height and weight) and sagittal parameters. P < 0.05 was considered to be statistically significant.

## Results

### General information

A total of 157 subjects were included in this study, comprising 52 subjects (14 males and 38 females) in the control group, 68 patients (11 males and 57 females) in the balance group, and 37 patients (7 males and 30 females) in the imbalance group. There was no significant difference in age, height, weight, BMI, and gender distribution among the three groups (P > 0.05) (Table 1).

**Table 1:** Demographic characteristics of three groups (mean ± SD)

Variable	All	Control group	Balance group	Imbalance group	P value
N	157	52	68	37	N/A
Gender(male/female)	32/125	14/38	11/57	7/30	0.351
Age(year)	63.6 ± 7.6	63.1 ± 5.6	62.6 ± 7.8	66.2 ± 9.3	0.060
Height(cm)	154.1 ± 7.5	155.9 ± 7.6	153.7 ± 7.7	152.4 ± 6.7	0.120
Weight(kg)	59.0 ± 8.7	59.0 ± 7.7	60.3 ± 9.1	56.6 ± 8.8	0.061
BMI(kg/m^2^)	24.8 ± 3.1	24.3 ± 2.6	25.5 ± 3.4	24.3 ± 3.2	0.055

**No significant differences between groups (p > 0.05)**

***Abbreviations***: **SD Standard deviation, BMI Body mass index**

### Comparison of MVE/ET of paraspinal muscles and sagittal parameters

MVE and ET of paraspinal muscles in the control group were significantly higher than those in the balance and imbalance groups (P < 0.05), whereas MVE in the balance group was significantly higher than that in the imbalance group (P < 0.05). SVA in the imbalance group was significantly higher than those in the control and balance groups (P < 0.05). SS and TK in the control group were significantly higher than those in the imbalance group (P < 0.05). PT and PI in the control group were significantly lower than those in the balance and imbalance groups (P < 0.05). LL in the imbalance group was significantly lower than that in the control and balance groups (P < 0.05) (Table 2).

**Table 2:** Strength/Endurance of Paraspinal Muscles and Sagittal Parameters of three groups(mean ± SD)

Parameters	Control group	Balance group	Unbalance group	P value			
				A vs. B vs. C	A vs. B	A vs. C	B vs. C
Muscle function							
MVE(N)	139.4 ± 37.1	103.8 ± 44.4	84.5 ± 44.5	P<0.001	P<0.001	P<0.001	0.026
ET(s)	71.5 ± 46.0	41.0 ± 31.8	33.6 ± 50.4	P<0.001	0.001	P<0.001	0.099
Sagittal parameter							
SVA(cm)	2.0 ± 1.4	2.3 ± 1.2	8.9 ± 4.1	P<0.001	0.989	P<0.001	P<0.001
SS(°)	34.6 ± 8.7	30.3 ± 12.2	27.6 ± 12.1	0.007	0.092	0.007	0.613
PT(°)	13.3 ± 7.0	24.2 ± 9.8	30.3 ± 11.9	P<0.001	P<0.001	P<0.001	0.104
PI(°)	47.9 ± 12.3	54.5 ± 13.2	57.9 ± 14.9	0.001	0.008	0.002	1.000
LL(°)	50.0 ± 10.6	43.9 ± 20.1	27.9 ± 19.5	P<0.001	0.106	P<0.001	P<0.001
TK(°)	30.3 ± 8.9	31.0 ± 17.1	23.8 ± 12.7	0.030	1.000	0.040	0.072

***Abbreviations***: **SVA sagittal vertical axis, TK thoracic kyphosis, LL lumbar lordosis, PI pelvic incidence, PT pelvic tilt, SS sacral slope, MVE maximal voluntary exertion, ET Endurance time, A Control group, B Balance group, C Unbalance group**

### Correlation between MVE/ET of paraspinal muscles and sagittal parameters

**Fig. 2:**
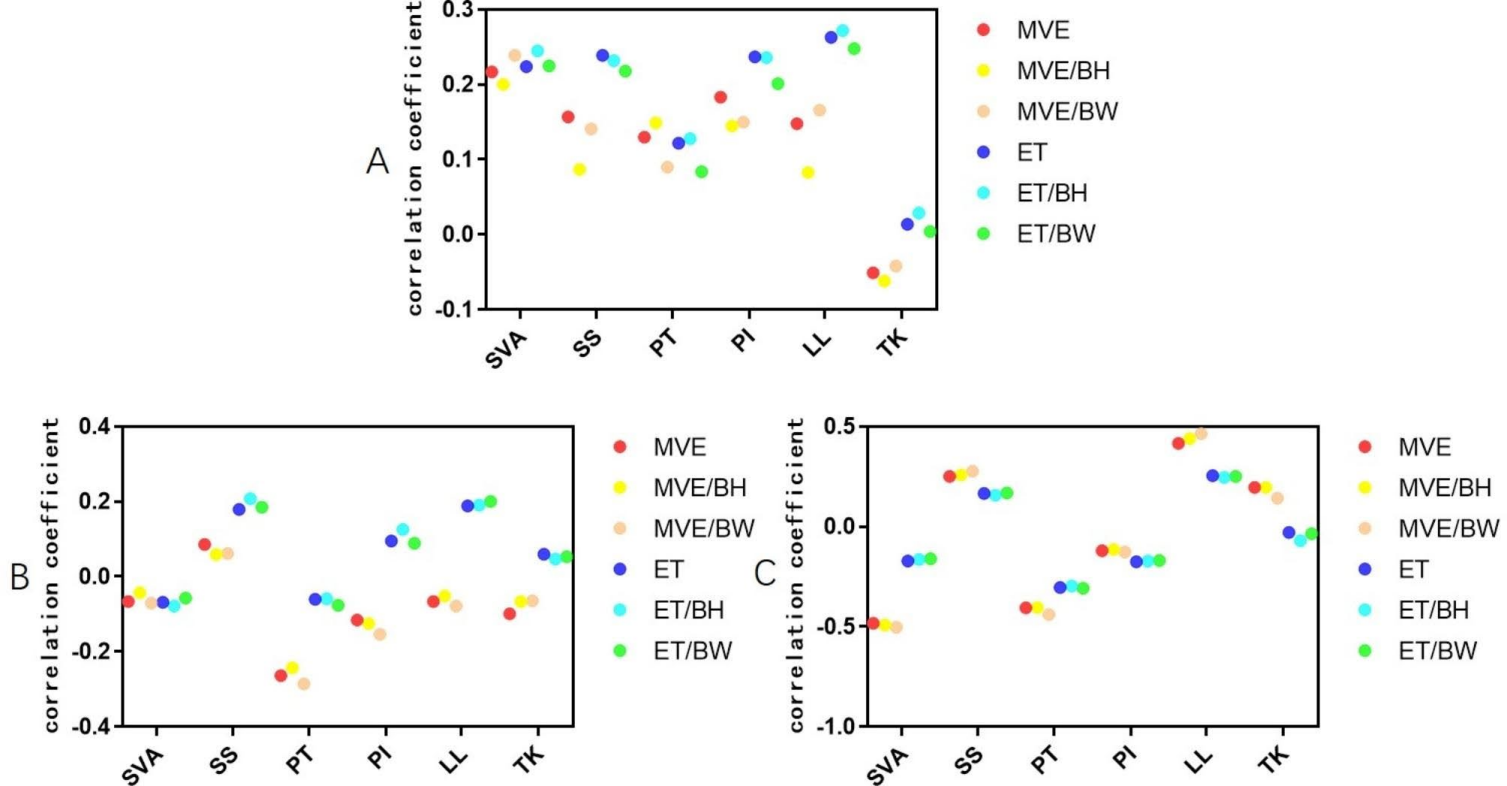
Scatter plot of correlation coefficient between MVE/ET of paraspinal muscles in the prone position and sagittal parameters. Illustration: (**A**) There was no significant correlation between the MVE/ET of paraspinal muscles and sagittal parameters in the control group. (**B**) There was a significant negative correlation between MVE, MVE/BH, MVE/BW, and PT in the balance group. (**C**) The MVE, MVE/BH, and MVE/BW of paraspinal muscles in the imbalance group were negatively correlated with SVA and PT and positively correlated with LL. SVA, sagittal vertical axis; TK, thoracic kyphosis; LL, lumbar lordosis; PI, pelvic incidence; PT, pelvic tilt; SS, sacral slope; MVE, maximal voluntary exertion; ET, endurance time; BH, body height; BW, body weight

There was no significant correlation between the MVE/ET of paraspinal muscles and sagittal parameters in the control group (P > 0.05) (Table 3; Fig. 2). There was a significant negative correlation between MVE, MVE/BH, MVE/BW, and PT in the balance group (r = − 0.264, − 0.243, − 0.286, P < 0.05), but no significant correlation with other parameters (P > 0.05). There was no significant correlation between the ET of paraspinal muscles and sagittal parameters in the balance group (P > 0.05) (Table 4; Fig. 2). MVE, MVE/BH, and MVE/BW of paraspinal muscles in the imbalance group were negatively correlated with SVA (r = − 0.483, − 0.492, − 0.503, P < 0.05), negatively correlated with PT (r = − 0.405, − 0.404, − 0.439, P < 0.05), positively correlated with LL (r = 0.416, 0.439, 0.464, P < 0.05), but not correlated with other parameters (P > 0.05). No significant correlation was found between paraspinal muscle endurance and sagittal parameters in the imbalance group (P > 0.05) (Table 5; Fig. 2).

**Table 3:** Correlation analysis between MVE/ET of paraspinal muscles and sagittal parameters in control group

Parameters	SVA	SS	PT	PI	LL	TK
MVE	0.217	0.157	0.130	0.183	0.148	-0.051
MVE/BH	0.200	0.087	0.149	0.145	0.083	-0.062
MVE/BW	0.239	0.141	0.090	0.150	0.166	-0.042
ET	0.224	0.239	0.122	0.237	0.263	0.014
ET/BH	0.245	0.232	0.128	0.236	0.272	0.029
ET/BW	0.225	0.218	0.084	0.201	0.248	0.004

***Abbreviations***: **SVA sagittal vertical axis, TK thoracic kyphosis, LL lumbar lordosis, PI pelvic incidence, PT pelvic tilt, SS sacral slope, MVE maximal voluntary exertion, ET Endurance time, BH body hight, BW body weight**

P < 0.05, **P < 0.01, matched analysis

**Table 4:** Correlation analysis between MVE/ET of paraspinal muscles and sagittal parameters in balance group

Parameters	SVA	SS	PT	PI	LL	TK
MVE	-0.066	0.086	-0.264*	-0.116	-0.066	-0.099
MVE/BH	-0.043	0.059	-0.243*	-0.125	-0.052	-0.066
MVE/BW	-0.070	0.062	-0.286*	-0.154	-0.078	-0.064
ET	-0.068	0.179	-0.060	0.095	0.188	0.060
ET/BH	-0.078	0.208	-0.059	0.126	0.190	0.047
ET/BW	-0.057	0.185	-0.076	0.089	0.200	0.053

***Abbreviations***: **SVA sagittal vertical axis, TK thoracic kyphosis, LL lumbar lordosis, PI pelvic incidence, PT pelvic tilt, SS sacral slope, MVE maximal voluntary exertion, ET Endurance time, BH body hight, BW body weight**

P < 0.05, **P < 0.01, matched analysis

**Table 5:** Correlation analysis between MVE/ET of paraspinal muscles and sagittal parameters in imbalance group

Parameters	SVA	SS	PT	PI	LL	TK
MVE	-0.483**	0.251	-0.405*	-0.121	0.416*	0.195
MVE/BH	-0.492**	0.258	-0.404*	-0.115	0.439**	0.196
MVE/BW	-0.503**	0.277	-0.439**	-0.127	0.464**	0.142
ET	-0.172	0.165	-0.305	-0.176	0.254	-0.029
ET/BH	-0.165	0.157	-0.297	-0.172	0.246	-0.070
ET/BW	-0.161	0.168	-0.308	-0.170	0.250	-0.036

***Abbreviations***: **SVA sagittal vertical axis, TK thoracic kyphosis, LL lumbar lordosis, PI pelvic incidence, PT pelvic tilt, SS sacral slope, MVE maximal voluntary exertion, ET Endurance time, BH body hight, BW body weight**

P < 0.05, **P < 0.01, matched analysis

## Discussion

The role of paraspinal muscles in spinal dynamic stability has gained wide attention by different research groups around the world [13, 14]. Strength and endurance are important functional physical indexes to assess muscle condition [15]. Poor trunk muscle strength is closely related to daily activities and functional performance in the elderly population and is one of the reasons for their increased risk of falls [16]. In addition, a decrease in muscle endurance is an important risk factor for lower back pain [17]. Angela [18]et al. believe that insufficient paraspinal muscle endurance is closely related to the occurrence of dysfunction in populations with chronic non-specific lower back pain. Therefore, it is crucial to evaluate the strength and endurance of the paraspinal muscles accurately and objectively. Previous studies have shown that the prone paraspinal muscle strength test has high reliability and validity [19], and researchers recommend it as the preferred method for clinical paraspinal muscle strength assessment. Han [20]et al. used prone position test devices to assess paravertebral muscle endurance, and they proposed a new functional grading of paravertebral muscle on this basis. Therefore, the device for testing the MVE and ET of paraspinal muscles in the prone position has the advantages of high safety and recognition, convenient assembly and disassembly, and no limitation of site conditions.

The results of intergroup comparison showed that the MVE and ET of paraspinal muscles in patients were significantly lower than those in healthy controls, which may result from the decrease of motor units and muscle fibers caused by paraspinal muscle atrophy in patients with DSD, thus resulting in insufficient generation of muscle strength [21]. Previous studies have shown [22] that the paraspinal muscles of patients with DSD have obvious fat deposition and sparse distribution of type I slow-twitch fibers, which leads to the decrease of muscle fiber recruitment ability, and finally shows the loss of paraspinal muscle endurance. Therefore, the attenuation of the strength and endurance of paravertebral muscles may be the result of the shape of the deformity itself. In addition, the strength of paravertebral muscles in the imbalance group was significantly lower than that in the balance group; however, the endurance duration was not significantly different, thus suggesting that the further attenuation of paravertebral muscle strength after deformity development may be an indirect cause of sagittal imbalance in patients with degenerative spinal deformities, which may affect their quality of life [23, 24]. In clinical practice, therefore, it is of high significance to prevent the occurrence of decompensation by appropriate functional exercise of the lumbar back muscles to improve muscle strength in patients with DSD that have not yet lost balance.

The results of this study showed that PT in patients with degenerative spinal deformity was significantly higher than that in controls, thus suggesting that patients included in this study had initiated the compensatory mechanism to maintain the trunk balance. However, there was no significant difference in PT between the imbalance group and balance group. A possible reason might be that the compensatory potential of the pelvis was close to the limit state before transition to the imbalance state in patients in the imbalance group, and the posterior tension provided by the paraspinal muscles was further reduced, resulting in imbalance. In addition, TK and LL in the imbalance group were significantly lower than those in the control group, which may be the final result of the compensatory mechanism initiated by the body to maintain trunk dynamic balance. The loss of LL is one of the initiating factors for the compensatory mechanism [25]. In the process of entering the imbalance state, the body maintains the center of gravity within the range of “economic cone” by reducing thoracic kyphosis and pelvic retroversion [26]. It was also found in this study that the PI of the deformity group was significantly higher than that of the control group. Previous studies have shown that high PI is a risk factor for degenerative spinal deformity and sagittal imbalance [27], and it may be related to sacroiliac joint torsion [28]. When the center of gravity of the human body moves forward due to the gradual imbalance of the sagittal position, the arm of force with the sacroiliac joint as fulcrum will increase accordingly, and at the same time, the shear force borne by the sacroiliac joint surface will increase correspondingly. To reduce the damage of shear force to joint surface, the sacrum may rotate forward gradually under the weakened posterior muscle strength, which is manifested as the increase of PI [29].

SVA represents the overall balance of the spine [2], which is affected by many factors, including the pelvic and thoracic compensatory factors, and the lower limbs. In this study, paravertebral muscle strength was positively correlated with SVA in the imbalance group. Previous studies showed that the decrease of trunk muscle mass and fatty change are closely related to the increase of SVA in the elderly population [30, 31], which is basically consistent with the results of the current study. Therefore, in patients with imbalance, sagittal imbalance may mean progressive attenuation of paraspinal muscle strength. In addition, this study showed that the strength of the paraspinal muscles was negatively correlated with PT, thus suggesting that the decline in paraspinal muscle strength may participate in the compensatory mechanism during the process of spinal deformity progression. In patients with imbalance, it was found that the strength of paraspinal muscles was positively correlated with LL. Wang [32]et al. argued that the total and functional cross-sectional areas of paraspinal muscles are proportional to lumbar lordosis, and a larger muscle cross-sectional area often predicts a better muscle strength [33, 34], which is consistent with the results of this study. To reduce the individual difference of different height and weight populations [35, 36], the correlation analysis between the measured paravertebral muscle MVE/ET and the sagittal parameters was repeated after weight correction. The results showed that the correlation between the weight-weighted paravertebral muscle MVE and the above sagittal parameters was further improved, suggesting that for patients with imbalance, the paravertebral muscle MVE/BW was more sensitive and effective in reflecting the overall imbalance degree, compensation degree, and lumbar lordosis size. This provides a new alternative index to evaluate the degree of compensation and imbalance for patients who cannot complete full-length spine imaging due to hip arthropathy, pelvic supination difficulties, and lower limb diseases. In addition, relative muscle strength is the preferred indicator for measuring muscle quality [37], and Man [38] also used the ratio of grip strength to arm mass as an evaluation indicator for arm muscle quality in his research. Therefore, the MVE/BW of paraspinal muscles may have important clinical significance in predicting paraspinal muscle quality.

This study had some limitations. First, it was a single-center study with a limited sample size. Second, this study did not focus on the correlation between paraspinal muscle degeneration and sagittal parameters and health-related quality of life. Therefore, large-sample dynamic observation is required in the future.

## Conclusions

The decrease of MVE and ET of paraspinal muscles in the prone position in patients with DSD may result from deformity. The decline in MVE of paraspinal muscles in the prone position may be a predisposing factor for the imbalance in patients with DSD. In patients with DSD, the decrease of MVE/BW of paraspinal muscles in the prone position may be involved in spinal compensation, and it is also a sensitive indicator for sagittal imbalance and lumbar lordosis.

## Data Availability

The datasets used and/or analyzed during the current study are available from the corresponding author on reasonable request.

## Abbreviations

DSD: degenerative spinal deformity
SVA: sagittal vertical axis
TK: thoracic kyphosis
LL: lumbar lordosis
PI: pelvic incidence
PT: pelvic tilt
SS: sacral slope
MVE: maximal voluntary exertion
ET: endurance time
